# Five-year prospective outcomes of medical management and microvascular decompression in trigeminal neuralgia

**DOI:** 10.1007/s00415-025-13447-9

**Published:** 2025-10-16

**Authors:** Jacob Worm, Tone Bruvik Heinskou, Per Rochat, Jacob Bertram Springborg, Emil Andonov Smilkov, Lars Bendtsen, Henrik Winther Schytz, Stine Maarbjerg

**Affiliations:** 1 Department of Neurology, Danish Headache Center, Copenhagen University Hospital – Rigshospitalet, Valdemar Hansens Vej 5, 2600 Glostrup, Denmark; 2 Department of Clinical Medicine, Faculty of Health and Medical Sciences, University of Copenhagen, Copenhagen, Denmark; 3 Department of Neurology, Neuropathic Pain and CRPS Clinic, Copenhagen University Hospital – Rigshospitalet, Glostrup, Denmark; 4 Department of Neurosurgery, Copenhagen University Hospital – Rigshospitalet, Copenhagen, Denmark; 5 Department of Radiology, Copenhagen University Hospital – Rigshospitalet, Glostrup, Denmark; 6 Department of Brain and Spinal Cord Injury, Copenhagen University Hospital – Rigshospitalet, Glostrup, Denmark

**Keywords:** Facial pain, Sex differences, Cohort studies, MVD, Pain management, Observational study, Neurosurgery, Anticonvulsants, MDT, Multidisciplinary

## Abstract

**Background:**

Trigeminal neuralgia is a severe facial pain disorder with major impact on quality of life. Long-term prospective data comparing medical and surgical treatments remain scarce. This study compared 5-year outcomes of medical management and microvascular decompression (MVD) and explored predictors of treatment response.

**Methods:**

We conducted a prospective observational cohort study at a national referral center (2012–2019). Consecutive patients with classical or idiopathic trigeminal neuralgia underwent MRI, baseline assessment, and systematic 5-year follow-up. Treatment allocation (medical therapy or MVD) followed clinical evaluation and international guidelines. The primary outcome was categorized as pain-free without medication, pain-free with medication, continued pain without medication, or continued pain with medication.

**Results:**

Of 626 eligible patients, 227 completed follow-up (95 MVD, 132 medical). Pain-free without medication was achieved in 59% of MVD patients versus 19% of medically treated [relative risk (RR) 3.03, 95% confidence interval (CI) 1.67–5.50]. Pain-free with medication occurred in 1% after MVD versus 14% of the medical cohort (RR 0.16, 95% CI 0.04–0.69). Continued pain on medication was less frequent after MVD (22% vs 49%; RR 0.44, 95% CI 0.24–0.80). Male sex predicted excellent outcome after MVD (odds ratio 2.60, 95% CI 1.02–6.91). Postoperative hypoesthesia occurred in 23% and hearing impairment in 7%, while 55% of medically treated patients reported side effects.

**Conclusions:**

MVD provided superior long-term pain relief, higher rates of medication-free pain freedom, and reduced medication use compared with medical management, supporting earlier surgical consideration in selected patients and emphasizing individualized, guideline-based care.

**Supplementary Information:**

The online version contains supplementary material available at 10.1007/s00415-025-13447-9.

## Introduction

Trigeminal neuralgia is a severe facial pain disorder characterized by recurrent, shock-like paroxysms affecting one or more branches of the trigeminal nerve [1]. It significantly impacts quality of life, with up to 45% of patients missing daily activities and more than 50% experiencing anxiety [2–5]. Trigeminal neuralgia also poses a substantial economic burden through direct medical costs [6] and productivity loss [7]. The management traditionally involves several specialties, including neurology, neurosurgery, radiology, and dentistry. Yet very few high-quality prospective studies evaluate treatment efficacy and side effects [8–11], and none compare medical and surgical outcomes.

First-line treatment consists of the sodium channel blockers carbamazepine and oxcarbazepine, while second-line options include gabapentin, pregabalin, and lamotrigine [8]. In a prospective study of 103 medically treated patients, 51% achieved more than 50% pain reduction after 2 years [12]. However, side effects are common, affecting 85%, with 44% discontinuing treatment due to intolerance [13–15]. Women may experience higher pain recurrence and lower tolerability to first-line drugs [16, 17], though the impact of sex on long-term outcomes remains unclear.

When medical treatment fails, international guidelines recommend procedures, such as microvascular decompression (MVD), balloon compression, or radiofrequency thermocoagulation [8, 18]. These options carry surgical risks [9, 19, 20], particularly MVD, which is the preferred option for classical trigeminal neuralgia where neurovascular contact (NVC) causes morphological changes of the trigeminal nerve, such as displacement, indentation, or atrophy [8]. MVD may also benefit some patients with idiopathic trigeminal neuralgia and NVC *without* morphological changes [9].

Long-term prospective data on medical and surgical outcomes, including prognostic factors, are essential to guide treatment decisions. Building on previous research [9, 12, 21, 22], this study aims to evaluate 5-year outcomes of medical treatment and MVD in trigeminal neuralgia patients managed within the same clinical setting. Objectives are to assess pain relief, medication use, and patient-reported outcomes, identify predictors of treatment success or failure, and, for the first time, directly compare long-term medical and surgical outcomes under standardized care.

## Methods

### Study design and population

This prospective, observational cohort study was conducted at the Danish Headache Center, a tertiary referral center for headache and facial pain. Patients with idiopathic or classical trigeminal neuralgia were enrolled in a structured multidisciplinary management program between May 2012 and December 2019 and provided informed consent [23]. Sample size was defined by database inclusion.

Treatment decisions, including referral for psychological and/or physiotherapy support, were made jointly by neurologist and patient in accordance with international guidelines [8]. All patients included in the MVD cohort had received medical treatment prior to surgical referral. Diagnosis followed the International Classification of Headache Disorders (ICHD), using the 3rd edition [1], the ICHD-3 beta [24], or the ICHD-2 [25], depending on the inclusion date.

Eligibility required a standardized 3.0 T MRI, baseline interview, neurological exam, and completion of the 5-year follow-up questionnaire. For the medical cohort, exclusion criteria were any trigeminal neuralgia surgery within the 5-year period, language barriers, or psychiatric illness affecting consent. Exclusion criteria for the MVD cohort included previous MVD, other surgical interventions for trigeminal neuralgia, language barriers, or psychiatric illness precluding informed consent.

### Data acquisition

Upon obtaining consent, baseline data were obtained from consecutively recruited patients, ensuring the absence of selection bias, through semi-structured interviews conducted by experienced neurologists or trained fellows with expertise in facial pain. Pain intensity was rated using a 0–10 Numeric Rating Scale (NRS), reflecting the last month’s average burden. A neurological exam focusing on facial sensory function was performed. Age and sex were obtained via national identification numbers. Race and ethnicity were not collected. After 5 years (post-MVD or post-initial visit, ± 6 months), patients completed a self-administered survey (see Supplement). Additional methodological details are available in previous publications [12, 21, 23].

### MRI protocol and definitions

Preoperative 3.0 T MRI was performed using a standardized protocol including whole-brain T2-weighted turbo spin-echo, thin-section T2-weighted GRASE sequences of the brainstem/posterior fossa, 3D balanced fast field echo (BFFE), and time-of-flight MR angiography (s3DI MC HR). Multiplanar reconstructions visualized the cisternal trigeminal nerve and NVC in axial, sagittal, and coronal planes. All scans were reviewed by a neuroradiologist (E.S. or F.W.), blinded to the symptomatic side, using predefined criteria. NVC was defined as vessel contact with the trigeminal nerve without visible cerebrospinal fluid. It was graded as simple contact or contact with morphological changes (i.e., compression, displacement, distortion, indentation, and/or atrophy), and further classified by vessel type (artery, vein, or mixed) and location (root entry zone or peripheral) [26].

### Surgical technique

MVD was performed via a modified Jannetta retrosigmoid approach [27]. Arterial compressions were treated by transposing the vessel and fixing it to the tentorium with glue and Teflon, or by interposing Teflon between the nerve and vessel when transposition was not possible. Venous compressions were often left untouched if there was an obvious arterial NVC. In some cases, veins were transposed or coagulated and divided. Perioperative findings were recorded in a standardized scheme by the treating neurosurgeon (P.R, J.S, or J.B) from the Department of Neurosurgery, Copenhagen University Hospital—Rigshospitalet. Further technical details have been described previously [9, 21].

### Outcome measures

Surgical and medical outcomes were assessed via self-report and independently evaluated by a physician from the research unit, which is based at the DHC but operates separately from the outpatient clinic to ensure impartiality. The primary outcome at 5-year follow-up was categorized into four predefined groups: pain-free without medication (excellent outcome), pain-free with medication, continued pain without medication, and continued pain with medication.

Secondary outcomes included predictors of the primary outcome, median pain reduction, ≥ 30% and ≥ 50% pain reduction, treatment satisfaction, depressive symptoms, surgical complications, medical treatment and side effects, and sex-stratified analyses of medication use and treatment response.

### Definitions of complications

Complications were self-reported as symptoms, reflecting patients’ perceived experiences rather than formal diagnoses. Facial hypoesthesia was reported if new or worsened postoperatively, including onset, severity, and whether spontaneous or touch-evoked. Hearing loss was graded as mild or severe, and described as remitted, remitting, unchanged, or worsened. Visual impairment was based on six predefined symptoms, but only diplopia was included due to limited reliability of the others. Facial palsy was recorded only when difficulty chewing, drooling, and biting the inner cheek co-occurred. Stroke-related symptoms (e.g., cranial nerve deficits, dizziness, and ataxia) were not listed separately, consistent with prior definitions [9].

### Statistical analyses

Analyses were conducted in R (v4.4.2). Categorical and continuous variables were compared using Chi-square or Fisher’s exact tests, and t tests or Wilcoxon rank-sum tests, as appropriate. Normality was assessed visually. Changes in pain over time were evaluated using Wilcoxon signed-rank test, and McNemar’s test was applied for binary outcomes. Proportions were assessed with binomial tests; 95% confidence intervals (CIs) were reported where relevant. Relative risk (RR) with 95% CIs were calculated to compare outcome proportions between groups. Missing data were excluded. Significance was set at *p* < 0.05.

Logistic regression with backward elimination identified predictors of excellent outcome. Covariates included sex, age, disease duration, NVC with morphological changes, hypertension, anxiety/depression, primary headache, concomitant trigeminal pain, and other chronic pain. In the medical cohort, additional models examined ≥ 30% and ≥ 50% pain reduction. Results are reported as odds ratios (ORs) with 95% CIs. Secondary and subgroup analyses were exploratory.

### Ethics approval and consent to participate

The Danish National Committee on Health Research Ethics reviewed both project protocols, H-16019808 and H-1-2012-093, and confirmed that ethical approval and individual patient consent were not required, as both studies were non-interventional, observational in nature, and based on routine clinical care and laboratory procedures. Nonetheless, written informed consent was obtained from all participants in accordance with the Danish Data Protection Agency’s regulations. The reporting of study findings followed the STROBE guidelines.

## Results

**Fig. 1:**
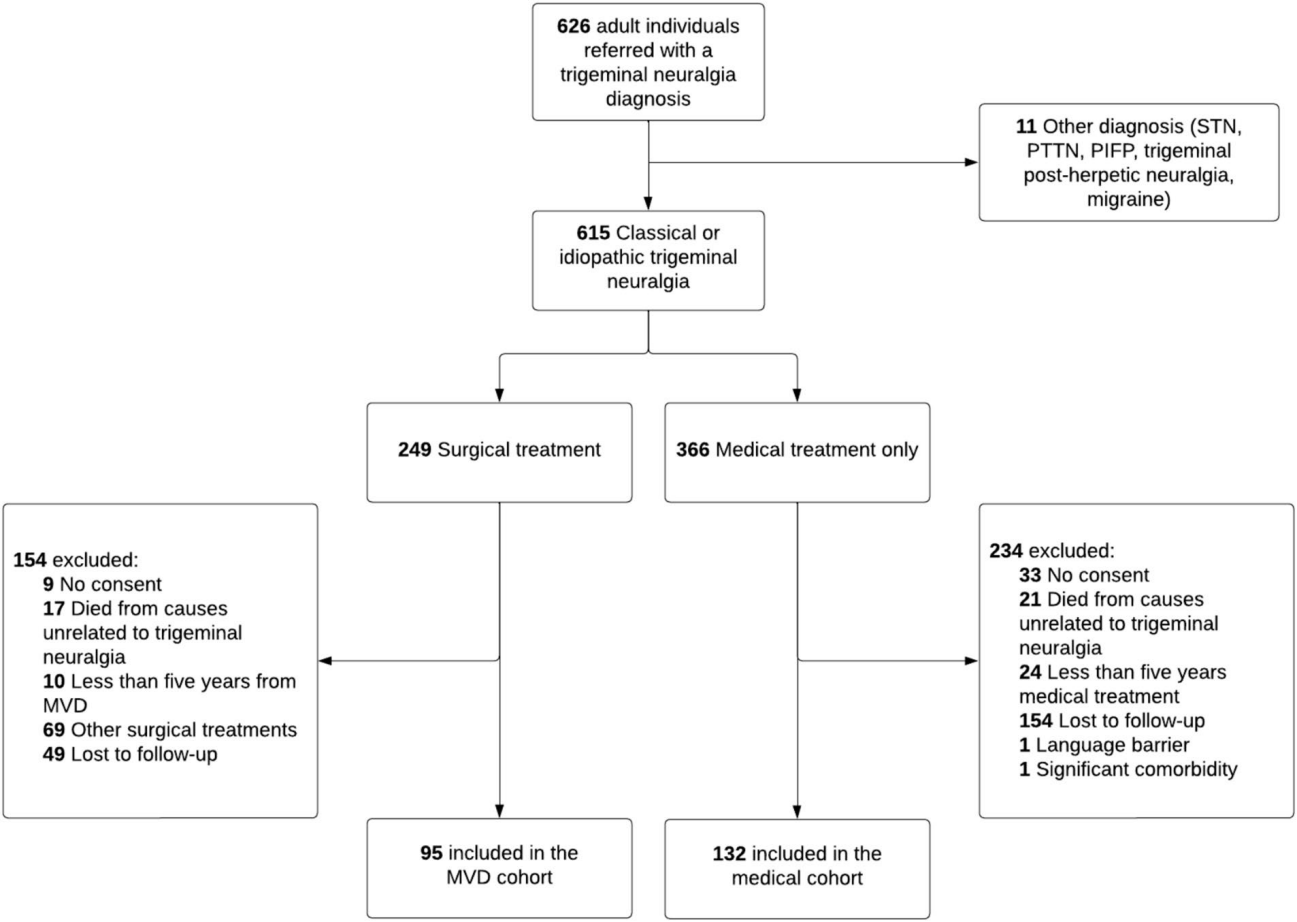
Flowchart of inclusion. Individuals with classical and idiopathic trigeminal neuralgia were divided into two groups based on whether they were medically managed or underwent microvascular decompression. *MVD* microvascular decompression, *STN* secondary trigeminal neuralgia, *PTTN* post-traumatic trigeminal neuropathy, *PIFP* persistent idiopathic facial pain

Between May 2012 and December 2019, 615 patients with trigeminal neuralgia were enrolled. At 5-year follow-up, 227 responded: 95 from the MVD cohort and 132 from the medical cohort (Fig. 1).

Mean age in the MVD cohort was 67.4 years [standard deviation (SD) 12.5] and 66.2 years (SD 13.5) in the medical cohort. Median disease duration was 9 years [interquartile range (IQR) 7] and 8 years (IQR 7), respectively. The cohorts were largely comparable, except for a higher proportion of men (45% vs 28%) and more frequent NVC with morphological changes (74% vs 48%) in the MVD cohort (Table 1).

**Table 1:** Demographics, clinical characteristics, and comorbidities

	MVD	Medical	Total
Demographics			
Total number of patients, *N* (%)	95	132	227
Women, *N* (%)	52 (55)	95 (72)	147 (65)
Men, *N* (%)	43 (45)	37 (28)	80 (35)
Current age, mean (SD), year	67.3 (12.5)	66.2 (13.5)	66.7 (13.1)
Age at onset, mean (SD), year	56.4 (12.5)	55.0 (13.8)	55.6 (13.3)
Disease duration, median (IQR), year	9 (7.0)	8 (7.0)	8 (7.0)
Clinical characteristics			
Right-sided pain, *N* (%)	59 (62)	72 (55)	131 (58)
Left-sided pain, *N* (%)	35 (37)	53 (40)	88 (39)
Bilateral pain, *N* (%)	1 (1)^a^	7 (5)	8 (3)
Concomitant persistent pain, *N* (%)	52 (55)	72 (55)	124 (55)
NVC with morphological changes, symptomatic side, *N* (%)	70 (74)	48 (38)	118 (52)
Localization of pain			
V1, *N* (%)	2 (2)	5 (4)	7 (3)
V2, *N* (%)	12 (13)	28 (21)	40 (18)
V3, *N* (%)	18 (19)	19 (14)	37 (16)
V1 + V2, *N* (%)	14 (15)	6 (5)	20 (9)
V2 + V3, *N* (%)	34 (36)	51 (39)	85 (37)
V1 + V2 + V3, *N* (%)	15 (16)	23 (17)	38 (17)
Comorbidities			
Hypertension, *N* (%)	27 (29)	34 (26)	61 (27)
Other chronic pain conditions, *N* (%)	8 (8)	20 (15)	28 (12)
Depression and/or anxiety, *N* (%)	10 (11)	18 (14)	28 (12)
Cardiovascular disease, *N* (%)	10 (11)	9 (7)	19 (8)
Tension-type headache, migraine, or cluster headache, *N* (%)	24 (25)	28 (21)	52 (23)

Baseline characteristics of patients in the microvascular decompression (MVD) and medical cohorts, including sex distribution, age, disease duration, pain laterality, pain distribution, presence of concomitant persistent pain, neurovascular contact (NVC) with morphological changes, and selected comorbidities (obtained via medical history). Data are presented as *N* (%) unless otherwise specified

*MVD* microvascular decompression, *SD* standard deviation, *IQR* interquartile range, *NVC* neurovascular contact

^a^The patient had bilateral pain, but MVD was performed and evaluated only on the right side

### Pain and medication status

**Fig. 2:**
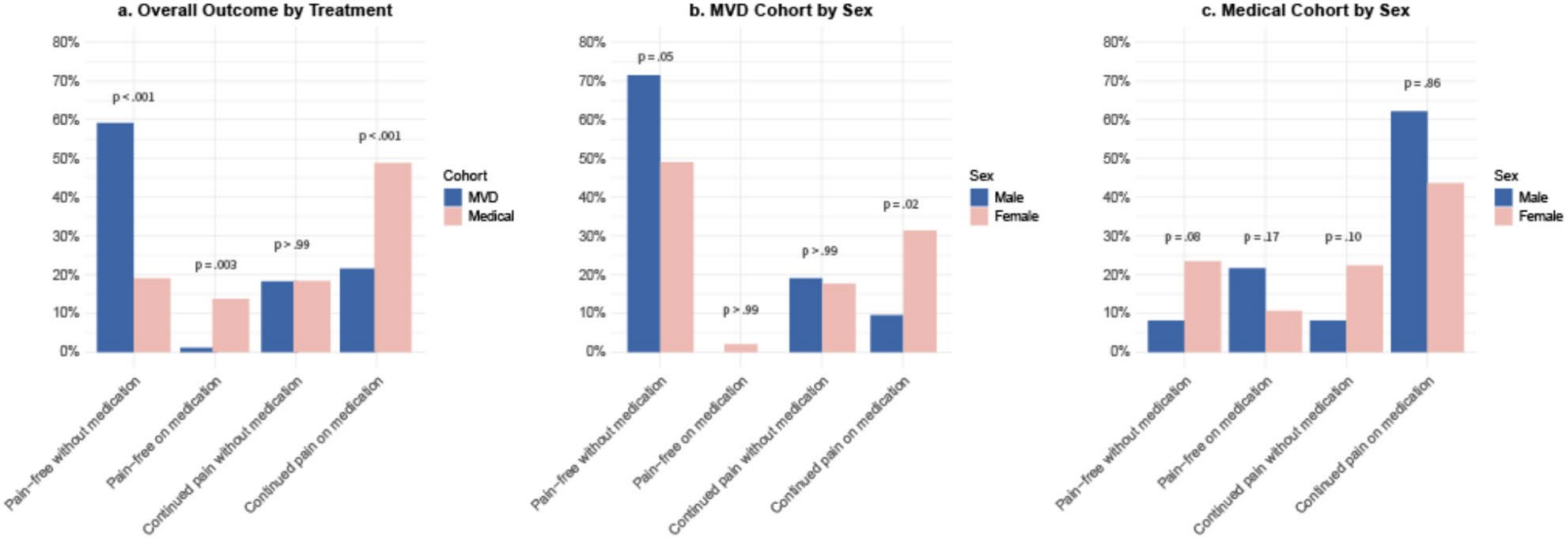
Pain and medication status by sex in the two cohorts at 5-year follow-up. Distribution of outcomes in the microvascular decompression (MVD) and medical cohorts across four predefined categories: pain-free without medication, pain-free on medication, continued pain without medication, and continued pain on medication (**a**). Sex-stratified distributions within the MVD (**b**) and medical (**c**) cohorts are also shown, with proportions for men and women in each outcome category, illustrating differences in long-term treatment response across sex and treatment modality

At 5 years, 59% of MVD patients were pain-free without medication, compared to 19% in the medical cohort (RR 3.03, 95% CI 1.67–5.50, *p* < 0.001). Pain freedom on medication was observed far less frequently in the MVD cohort (1%) compared with the medical cohort (14%) (RR 0.16, 95% CI 0.04–0.69, *p* = 0.003). Continued pain without medication was similar (18%) between groups (RR 1.06, 95% CI 0.54–2.08, *p* > 0.99), while continued pain on medication was less frequent in the MVD cohort (22%) than in the medical cohort (49%) (RR 0.44, 95% CI 0.24–0.80, *p* < 0.001) (Fig. 2a, Supplementary Table 1a).

### The overall burden of pain

Median NRS scores decreased from 7.0 to 0.0 in the MVD cohort and from 7.0 to 3.0 in the medical cohort (*p* = 0.004). A ≥ 50% NRS reduction was more common after MVD (65%) than with medical treatment (43%) (RR 1.89, 95% CI 1.19–3.01, *p* = 0.004). Similarly, a ≥ 30% pain reduction was more common in the MVD cohort (72%) than in the medical cohort (56%) (RR 1.80, 95% CI 1.80–2.70, *p* = 0.007). Pain freedom (NRS = 0), regardless of medication, was reported by 59% in the MVD cohort and 33% in the medical cohort (*p* < 0.001) (Table 2).

**Table 2:** Burden of pain and pain reduction at baseline and 5-year follow-up

	Baseline		5-Year		Between-group change from baseline to 5 years	*p* value
	MVD	Medical	MVD	Medical		
Numerical rating scale (NRS), median (IQR)	7 (4)^a^	7 (4)^c^	0 (5)^b^	3 (5)^d^	− 5 (6) vs. − 3 (5) (points)	< 0.001^1^ < 0.001^1^ 0.004^2^
≥ 50% pain reduction, *N* (%; 95% CI); RR (95% CI)	–	–	62 (65; 55–74)	57 (43; 35–52)	1.80 (1.20–2.70)	< 0.001^1^ 0.004^3^
≥ 30% pain reduction, *N* (%; 95% CI); RR (95% CI)	–	–	68 (72; 61–79)	74 (56; 48–64)	1.89 (1.19–3.01)	< 0.001^1^ 0.007^3^
No pain (NRS = 0), *N* (%; 95% CI)	3 (3; 1–9)	8 (6; 3–12)	56 (59; 49–68)	43 (33; 25–41)	+ 56 pp vs. + 27 pp	< 0.001^4^ < 0.001^4^ < 0.001^5^

Median pain intensity (NRS), proportion of patients achieving ≥ 30% and ≥ 50% pain reduction, and complete pain relief (NRS = 0) are shown for both cohorts at baseline and 5 years. Within-group changes were assessed using the Wilcoxon signed-rank^1^ and McNemar^4^ tests. Between-group comparisons used the Mann–Whitney *U* test^2^ and Chi-square tests^3,5^. Percentages are presented with 95% Wilson confidence intervals. ^a^Missing data from 3 patients; ^b^Missing data from 5 patients; ^c^Missing data from 4 patients; ^d^Missing data from 1 patient

*MVD* microvascular decompression, *IQR* interquartile range, *CI* confidence interval, *RR* relative risk, *pp* percentage points

### Predictors of excellent outcome

In the MVD cohort, men were more likely than women to be pain-free without medication at 5 years (71% vs 49%, *p* = 0.05) and less likely to report continued pain while on medication (10% vs 31%, *p* = 0.02). No significant sex differences were observed in the medical cohort (Fig. 2B + C, Supplementary Table 1B + C).

In multivariable analysis, male sex was associated with higher odds of an excellent outcome following MVD (OR 2.60, 95% CI 1.02–6.91, *p* = 0.05). A non-significant trend was observed for better outcomes in patients with NVC with morphological changes compared to those without such changes or with no identifiable NVC (OR 2.56, 95% CI 0.94–7.23, *p* = 0.07). No interaction between sex and NVC was observed (*p* = 0.51). No significant predictors were identified in the medical cohort for excellent outcome or for achieving ≥ 30% or ≥ 50% pain reduction (Supplementary Fig. 1a + b).

### Medication use and sex differences

At baseline, medication use was similar across both cohorts (80%, *p* > 0.99) (Table 3). At 5 years, fewer MVD patients remained on medical treatment compared to the medical cohort (23% vs 62%, *p* < 0.001).

**Table 3:** Medication use at baseline and 5-year follow-up

	Baseline					5 years				*p* value
	Medical (*N* = 132)		MVD (*N* = 95)			Medical (*N* = 132)		MVD (*N* = 95)		
	*N* (%)	95% CI	*N* (%)	95% CI	*p* value	*N* (%)	95% CI	*N* (%)	95% CI	
Medication-free	27 (21)	14–28	19 (20)	13–30	> 0.99	50 (38)	30–47	73 (77)	67–85	< 0.001
On medication	105 (80)	72–86	76 (80)	71–88	> 0.99	82 (62)	53–70	22 (23)	15–33	< 0.001
Carbamazepine	32 (31)	22–40	21 (28)	18–39		26 (32)	22–43	5 (23)	8–45	
Oxcarbazepine	28 (27)	19–36	22 (29)	19–41		27 (33)	23–44	8 (36)	17–59	
Gabapentin	36 (34)	25–44	22 (29)	19–41		24 (29)	20–40	6 (27)	11–50	
Pregabalin	13 (12)	7–20	15 (20)	12–31		11 (13)	7–23	1 (5)	0–23	
Lamotrigine	7 (7)	3–13	7 (9)	4–18		17 (21)	13–31	3 (14)	3–35	
Other	16 (15)	9–24	23 (30)	19–42		13 (16)	9–26	10 (46)	24–68	
First-line	60 (57)	47–67	43 (57)	45–68	> 0.99	53 (65)	53–75	13 (59)	36–79	0.82
Second-line	52 (50)	40–60	39 (51)	40–63	0.93	46 (56)	45–67	9 (41)	21–63	0.30
One drug	80 (76)	67–84	46 (61)	49–72		51 (62)	51–72	13 (59)	36–79	
Two drugs	23 (22)	14–31	27 (36)	25–47		26 (32)	22–43	7 (32)	14–55	
Three or more drugs	2 (2)	0–7	3 (4)	1–11		5 (6)	2–14	2 (9)	1–29	

Proportions and 95% confidence intervals are shown for each medication type, stratified by treatment group and time point. First-line medications include carbamazepine and oxcarbazepine; second-line include gabapentin, pregabalin, and lamotrigine. Comparison of medication-free status and first-line vs. second-line drug use between groups at each time point was assessed using Pearson’s *χ*^2^ test with Yates’ continuity correction

*MVD* microvascular decompression, *CI* confidence interval

In the MVD cohort, baseline medication use did not differ by sex (*p* = 0.64), but by 5 years, a higher proportion of men had discontinued treatment (91% vs 65%, *p* = 0.003). Use of second-line drug remained more common in women (0% vs 50%, *p* = 0.01) (Supplementary Table 2a).

In the medical cohort, baseline medication use was slightly higher among men (89% vs 76%, *p* = 0.10), and they more often used first-line drugs (73% vs 50%, *p* = 0.05). At follow-up, men were more likely to remain on medication (84% vs 54%, *p* = 0.003), but second-line drug use did not differ significantly between sexes (42% vs 53%, *p* = 0.43) (Supplementary Table 2b).

### Side effects in the medical cohort and surgical complications

MVD complications at 1 and 2 years have been reported previously [9, 21]. At 5 years, 21 patients (23%) reported persistent ipsilateral hypoesthesia, which was severe in 7 cases (7.4%). Additional complications included hearing loss in 7 patients (7.4%), diplopia in 6 (6.3%), facial palsy in 4 (4.2%), ischemic stroke in 3 (3.0%), and intracerebral hemorrhage in 1 (1.0%) (Table 4).

**Table 4:** Self-reported complications after microvascular decompression

Complication	During the first 12 months (*N* = 115)*	24-months follow-up (*N* = 115)*	5-year follow-up (*N* = 95)
	*N* (%)	*N* (%)	*N* (%)
Death	0	–	
Infarction; cerebellar or brainstem	6 (5)	6 (5)	3 (3)
Hemorrhage; cerebellar or brainstem	1 (1)	1 (1)	1 (1)
Anesthesia dolorosa	0^a^	0	–
Meningitis	0	0	–
Cerebrospinal fluid leak	6 (5)	0	–
Hydrocephalus	0	0	–
Ataxia	8 (7)^b^	6 (5)	md^#^
Diplopia	0^c^	0	6 (6.3)
Corneal keratitis	2 (2)^d^	2 (2)	
Severe hypoesthesia	8 (7)^e^	7 (6)	7 (7.4)
Any hypoesthesia	20 (18)	14 (12)	21 (23)
Facial weakness/facial nerve palsy	3 (3)^f^	3 (3)	4 (4.2)
Hearing loss	1 (1)	1 (1)	0
Hearing impairment	11 (10)	10 (9)	7 (7.4)

^*^Andersen et al. [9]

^a^One stroke patient had anesthesia dolorosa as a sequela after an infarct and reported by the patient

^b^All patients who suffered a stroke had permanent ataxia

^c^Three patients who suffered a stroke had permanent diplopia

^d^Two patients who suffered a stroke had corneal keratitis

^e^One patient who suffered a stroke had permanent hypoesthesia

^f^One patient who suffered a stroke had permanent facial weakness

^#^Not included in the 5-year questionnaire

Among 82 medical patients on medication, 33 (40%) reported no side effects, 23 (28%) had mild, 16 (20%) moderate, and 6 (7%) severe side effects. Four patients (5%) did not respond.

### Quality of life

Depressive symptoms were reported by 29 (31%) of MVD patients and 49 (39%) in the medical cohort (*p* = 0.26) at 5 years. Median satisfaction (0–7) was high: 7.0 in the MVD cohort, and 6.0–6.5 across efficacy, side effects, and information domains in the medical cohort. Nurse contact was more common than contact with physiotherapists or psychologists in both cohorts (Supplementary Table 3a + b). Overall, 84 (92%) of 95 MVD patients would recommend the procedure.

## Discussion

This 5-year study is the first to prospectively describe long-term outcomes of surgical (*n* = 95) and medical (*n* = 132) treatment for trigeminal neuralgia within the same clinical setting using guideline-adherent care [8]. With the highly important caveat that the two groups do not directly compare, both treatment pathways led to substantial pain reduction and high patient satisfaction. MVD, however, was associated with higher rates of pain freedom, greater overall pain reduction, and lower medication use, supporting earlier findings of its superiority [9, 28]. The evidence base for current medical treatment guidelines remains limited, ranging from very low to moderate quality, with few randomized placebo-controlled trials [8]. In this context, real-world studies like ours provide important evidence to guide treatment decisions.

### Long-term outcomes in medical vs surgical treatment

Both cohorts experienced a similarly high pain burden at baseline. At 5 years, nearly 60% of MVD patients were pain-free, compared to about one-third of the medical cohort. Notably, over half of medically treated patients achieved a ≥ 30% pain reduction—a threshold considered clinically meaningful [29–31]—exceeding rates typically observed in central neuropathic pain or diabetic neuropathy [32, 33]. These results support meaningful long-term benefit from both treatment strategies when delivered in a specialized, multidisciplinary setting [12, 34].

### Predictors of long-term outcome

In the MVD cohort, male sex was significantly associated with excellent surgical outcome, consistent with previous studies [9, 16, 21]. NVC with morphological changes showed a non-significant trend toward better outcomes, possibly reflecting limited power or biological heterogeneity. While male sex was independently associated with better outcomes, formal interaction analysis did not support a sex-specific effect of NVC. Our earlier work suggests that even patients with idiopathic trigeminal neuralgia and (simple) NVC *without* morphological changes may benefit from MVD, though these cases require individualized evaluation using high-resolution MRI [9]. The current findings support this as 26% of the MVD patients did not present with morphological changes on a preoperative MRI.

### Medication use across treatment pathways

MVD was linked to a substantial reduction in medication use. At 5 years, most surgical patients had discontinued medical treatment, while the medical cohort showed relatively stable medication use over time, with some patients continuing long-term combination therapy. The reduction in medication use following MVD might reflect its role in targeting the presumed root cause of classical trigeminal neuralgia, rather than symptom reduction [26, 35]. Importantly, medication discontinuation is likely to contribute to improved quality of life, given the side-effect burden observed in this study and in previous studies, of standard trigeminal neuralgia drugs [2, 15, 36]. Furthermore, long-term use of AED in trigeminal neuralgia patients might impair cognition [37].

### Sex-based differences in treatment and outcome

In the MVD cohort, men more frequently achieved pain freedom and discontinued medication, aligning with prior studies showing better surgical outcomes [9], lower recurrence risk [28], and greater medication tolerability [16] in men. Women more often continued treatment, typically with second-line drugs. In the medical cohort, men more frequently used first-line sodium channel blockers, while women tended to use second-line drugs. At follow-up, more men remained on medication, often using combination therapy.

These sex-based differences may reflect anatomical, hormonal, or neurophysiological factors [38]. Idiopathic trigeminal neuralgia, which tends to be less responsive to MVD [9], is more common in women [16], who are also more likely to report concomitant continuous pain [39], possibly linked to central sensitization [40]. Delayed referral in women, previously reported for Gamma Knife radiosurgery [41], may further contribute to poorer outcomes. These findings support personalized treatment and the development of better-tolerated options, including newer sodium channel agents [29], botulinum toxin A [42], and emerging therapies [43].

### Reconsidering the natural history of trigeminal neuralgia

Our findings challenge the view of trigeminal neuralgia as a progressive disorder [13, 44]. With the important caveat that the medical cohort did not include patients who were deemed medically refractory and therefore referred for surgery, the medical cohort demonstrated significant pain reduction, with few worsening over time. This supports previous real-world studies suggesting that, with appropriate medical management, patients with trigeminal neuralgia who respond to medical treatment may remain stable or even improve, rather than inevitably deteriorate [12, 34].

### Complications after microvascular decompression

Despite superior outcomes, MVD was associated with complications, consistent with prior studies using rigorous outcome reporting [9, 27, 28, 45–47]. However, self-reported complications without objective reassessment may overestimate their occurrence and persistence. Most complications were mild, and over 90% of patients would recommend the procedure. Nonetheless, MVD should be considered against less-invasive options, such as Gamma Knife radiosurgery, balloon compression, or radiofrequency ablation, which carry lower immediate risks but also lower success rates [19, 41].

### Psychosocial outcomes and support needs

Comorbid depression and/or anxiety was present in 11% of MVD patients and 14% of the medical cohort. Despite pain improvement, depressive symptoms persisted in one-third of MVD patients and nearly 40% of the medical cohort, exceeding rates in the general Northern European population where 34.5% of women and 20% of men reports depressive symptoms [48]. A few patients accessed physiotherapy or psychological services, which may help address musculoskeletal issues or chronic pain coping. Integrating mental health and rehabilitative support into trigeminal neuralgia care could improve outcomes. Given the substantial burden of trigeminal neuralgia, a multidisciplinary approach remains essential [6, 12, 36, 49].

### Clinical implications

Current guidelines reserve MVD for medically refractory trigeminal neuralgia, typically after failure of carbamazepine or oxcarbazepine and second-line options [8]. These findings support earlier surgical referral, particularly for patients with classical trigeminal neuralgia and identifiable NVC with morphological changes, and in selected cases of idiopathic trigeminal neuralgia. Standardized referral pathways and access to high-resolution imaging may facilitate more consistent, evidence-based treatment decisions.

### Study strengths, limitations, and future directions

A major strength of this study is its prospective design with standardized follow-up and real-world inclusion of both medical and surgical management pathways within a single tertiary care setting. Independent outcome assessments further enhance validity. However, the non-randomized treatment allocation may introduce selection bias, as patients with more severe pain were more likely to undergo MVD, which would most likely bias results against rather than in favor of surgical outcomes. Higher loss to follow-up in the medical cohort may introduce attrition bias [50], a known challenge in long-term trigeminal neuralgia studies [28, 36].

Another limitation is the reliance on self-reported outcomes in the medical cohort without independent verification. The study was not designed to reliably assess facial palsy or visual disturbances, as validated tools [51, 52] were not applied. Future studies should incorporate objective outcome assessments, standardized drug trials, and broader measures, such as attack frequency, functional impact, and quality of life [53–55].

## Conclusion

This prospective, real-world study strongly indicates that MVD provides superior long-term pain relief, pain freedom, and reduced medication use compared to medical therapy in trigeminal neuralgia. The male sex and the presence of NVC with morphological changes were associated with better surgical outcomes. These findings support earlier surgical consideration in selected patients and emphasize the importance of individualized, guideline-based treatment strategies.

## Supplementary Information

Below is the link to the electronic supplementary material.

Supplementary file1 (DOCX 70 KB)

## Data Availability

Jacob Worm, MD (Department of Neurology, Danish Headache Center, Rigshospitalet—Glostrup) conducted the statistical analysis. As corresponding author, he had full access to all the data in the study and takes responsibility for the integrity of the data and the accuracy of the data analysis. All coauthors meet authorship criteria and approve the final manuscript. Due to the rare nature of the condition and the limited number of participants, sharing individual-level data publicly could compromise confidentiality. Researchers who meet the criteria for access to confidential data may contact the corresponding author to request further details about the dataset.
