# Supplementary material for: Five-year prospective outcomes of medical management and microvascular decompression in trigeminal neuralgia
Source: J Neurol. 2025 Oct 16;272(10):701. doi: 10.1007/s00415-025-13447-9 (PMC12532645; doi:10.1007/s00415-025-13447-9)
Supplement: Supplementary file 1 — Supplementary file1 (DOCX 70 KB) [file 415_2025_13447_MOESM1_ESM.docx]

**Supplementary Material**

**Supplementary Figure 1a**


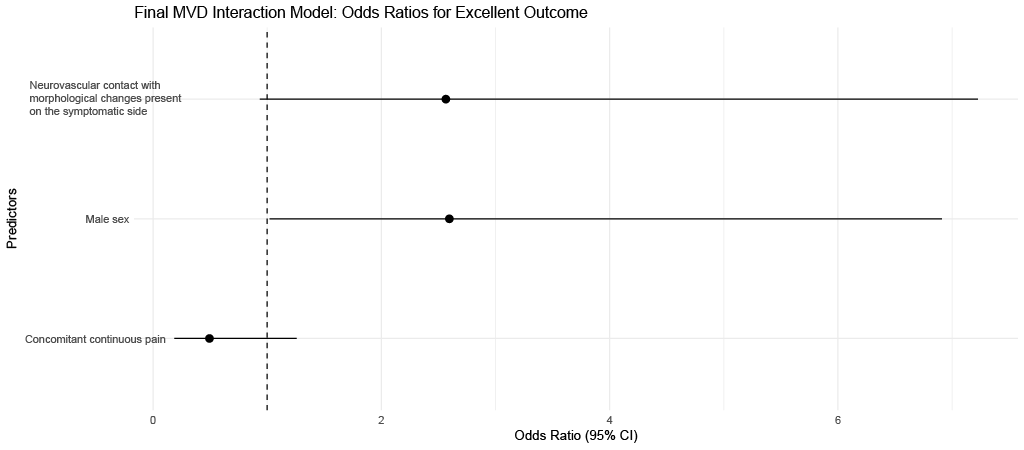


Final logistic regression model after backward elimination showing odds ratios (ORs) with 95% confidence intervals for predictors of excellent outcome (defined as pain freedom without medication) in the MVD cohort. The model was reduced from nine to three predictors: male sex (OR, 2.60; 95% CI 1.02-6.91; p = .05), neurovascular contact (NVC) with morphological changes (OR, 2.57; 95% CI 0.94-7.23; p = .07), and concomitant persistent pain (OR, 0.49; 95% CI 0.19-1.26; p = .14).

**Supplementary Figure 1b**


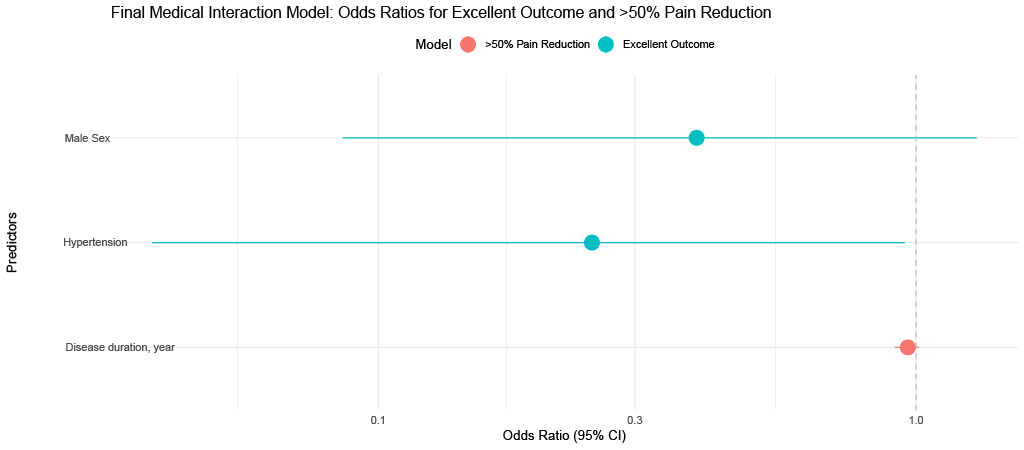


Final logistic regression model after backward elimination showing odds ratios (ORs) with 95% confidence intervals for predictors of excellent outcome (defined as pain freedom and no medication use), >50% or 30% pain reduction in the medically managed cohort. The models for excellent outcome retained two predictors: male sex (OR, 0.39; 95% CI 0.09-1.30; p = .16), hypertension (OR, 0.25; 95% CI 0.04-0.95; p = .08), and the model for >50% pain reduction only retained disease duration (OR, 0.97; 95% CI 0.91-1.01; p = .17), none of which were statistically significant and none were identified for >30% reduction.

| **Supplementary Table 1a. Pain and medication status After Five Years: Microvascular Decompression vs. Medical Management** | | | | | | | |
| --- | --- | --- | --- | --- | --- | --- | --- |
|  | **MVD** | | **Medical** | |  | | |
| **Outcome** | N (%) | 95% CI | N (%) | 95% CI | RR | 95% CI | *p*-value |
| **Pain-free without medication** | 55 (59) | 49-69 | 25 (19) | 13-27 | 3.03 | 1.67 – 5.50 | < .001 |
| **Pain-free on medication** | 1 (1) | 0-59 | 18 (14) | 8-21 | 0.16 | 0.04 – 0.69 | .003 |
| **Continued pain without medication** | 17 (18) | 11-28 | 24 (18) | 12-26 | 1.06 | 0.54 – 2.08 | > .99 |
| **Continued pain on medication** | 20 (22) | 14-31 | 64 (49) | 40-58 | 0.44 | 0.24 – 0.80 | < .001 |
| Distribution of patients across four predefined outcome categories at five years, comparing the MVD and medical cohorts. Values are shown as proportions with 95% confidence intervals (CI). Risk ratios (RR) and 95% CI were calculated using the medical cohort as reference. P-values reflect comparisons between groups using Fisher’s exact test or Pearson’s Chi-square test with Yates’ continuity correction. Abbreviations: MVD, microvascular decompression; CI, confidence interval; RR, relative risk. | | | | | | | |
| **Supplementary Table 1b. Sex-Stratified Pain and Medication Status After Five Years, Microvascular Decompression Cohort** | | | | | | | |
|  | **Men** | | **Women** | |  | | |
| **Outcome** | N (%) | 95% CI | N (%) | 95% CI | RR | 95% CI | *p*-value |
| **Pain-free without medication** | 30 (71) | 58-85 | 25 (49) | 35-63 | 1.45 | 0.62 – 3.41 | .05 |
| **Pain-free on medication** | 0 (0) | NA | 1 (2) | 0-6 | 0.40 | 0.02 – 10.2 | > .99 |
| **Continued pain without medication** | 8 (19) | 7-31 | 9 (18) | 7-28 | 1.09 | 0.39 – 3.08 | > .99 |
| **Continued pain on medication** | 4 (10) | 1-18 | 16 (31) | 19-44 | 0.33 | 0.11 – 1.03 | .02 |
| Sex-stratified outcomes in the MVD cohort at five years, categorized into four predefined groups. Proportions are presented with 95% CI. Risk ratios (RR) compare outcomes in women relative to men. P-values were calculated using Fisher’s exact test or Pearson’s Chi-square test with Yates’ continuity correction. Abbreviations: MVD, microvascular decompression; CI, confidence interval; RR, relative risk. | | | | | | | |
| **Supplementary Table 1c. Sex-Stratified Pain and Medication Status After Five Years, Medical Cohort** | | | | | | | |
|  | **Men** | | **Women** | |  | | |
| **Outcome** | N (%) | 95% CI | N (%) | 95% CI | RR | 95% CI | *p*-value |
| **Pain-free without medication** | 3 (8) | 0-17 | 22 (23) | 15-32 | 0.40 | 0.12 – 1.31 | .08 |
| **Pain-free on medication** | 8 (22) | 8-35 | 10 (11) | 4-17 | 2.06 | 0.76 – 5.57 | .17 |
| **Continued pain without medication** | 3 (8) | 0-17 | 21 (23) | 14-31 | 0.41 | 0.12 – 1.37 | .10 |
| **Continued pain on medication** | 23 (62) | 47-78 | 41 (44) | 34-54 | 1.44 | 0.67 – 3.11 | .86 |
| Sex-stratified outcomes in the medical cohort at five years across four predefined categories. Results include proportions with 95% CI, risk ratios (RR) comparing outcomes in women relative to men, and corresponding p-values from Fisher’s exact or Pearson’s Chi-square tests. Abbreviations: CI, confidence interval; RR, relative risk. | | | | | | | |

| **Supplementary Table 2a. Sex-Stratified Medication Use, MVD Cohort** | | | | | | | | | | |
| --- | --- | --- | --- | --- | --- | --- | --- | --- | --- | --- |
|  | **Baseline** | | | | *p*-value | **Five years** | | | | *p-*value |
|  | **Men** | | **Women** | |  | **Men** | | **Women** | |  |
|  | N (%) | 95% CI | N (%) | 95% CI |  | N (%) | 95% CI | N (%) | 95% CI |  |
| **Medication-free** | 10 (23) | 12-39 | 9 (17) | 8-30 | .64^2^ | 39 (91) | 78-97 | 34 (65) | 51-78 | .004^1^ |
| **On medication** | 33 (77) | 61-88 | 43 (83) | 70-92 |  | 4 (9) | 3-22 | 18 (35) | 22-49 |  |
| **Carbamazepine** | 13 (39) | 23-58 | 8 (19) | 8-33 |  | 2 (50) | 7-93 | 3 (17) | 4-41 |  |
| **Oxcarbazepine** | 9 (27) | 13-46 | 13 (30) | 17-46 |  | 1 (25) | 1-81 | 7 (39) | 17-64 |  |
| **Gabapentin** | 8 (24) | 11-43 | 14 (33) | 19-49 |  | 0 | 0 | 6 (33) | 13-59 |  |
| **Pregabalin** | 8 (24) | 11-42 | 7 (16) | 7-31 |  | 0 | 0 | 1 (6) | 0-27 |  |
| **Lamotrigine** | 2 (6) | 1-20 | 5 (12) | 4-25 |  | 0 | 0 | 3 (17) | 4-41 |  |
| **Other** | 8 (24) | 11-42 | 15 (35) | 21-51 |  | 2 (50) | 7-93 | 8 (44) | 22-69 |  |
| **First-line** | 22 (67) | 48-82 | 21 (49) | 33-65 | .19^2^ | 3 (75) | 19-99 | 10 (56) | 31-79 | .62^1^ |
| **Second-line** | 16 (48) | 31-67 | 23 (53) | 38-69 | .84^2^ | 0 | 0 | 9 (50) | 26-74 | .01^1^ |
| **One drug** | 19 (58) | 39-75 | 27 (63) | 47-77 |  | 3 (75) | 19-99 | 10 (56) | 31-79 |  |
| **Two drugs** | 13 (39) | 23-58 | 14 (33) | 19-49 |  | 1 (25) | 1-81 | 6 (33) | 13-59 |  |
| **Three or more drugs** | 1 (3) | 0-16 | 2 (5) | 1-16 |  | 0 | 0 | 2 (11) | 1-35 |  |
| **Supplementary Table 2b. Sex-Stratified Medication Use, Medical Cohort** | | | | | | | | | | |
|  | **Baseline** | | | | *p-*value | **Five years** | | | | *p-*value |
|  | **Men** | | **Women** | |  | **Men** | | **Women** | |  |
|  | N (%) | 95% CI | N (%) | 95% CI |  | N (%) | 95% CI | N (%) | 95% CI |  |
| **Medication-free** | 4 (11) | 3-25 | 23 (24) | 16-34 | .10^1^ | 6 (16) | 6-32 | 44 (46) | 36-57 | .003^2^ |
| **On medication** | 33 (89) | 75-97 | 72 (76) | 66-84 |  | 31 (84) | 68-94 | 51 (54) | 43-64 |  |
| **Carbamazepine** | 9 (27) | 13-46 | 23 (32) | 21-44 |  | 10 (33) | 17-51 | 16 (31) | 19-46 |  |
| **Oxcarbazepine** | 15 (46) | 28-64 | 13 (18) | 10-29 |  | 14 (45) | 27-64 | 13 (26) | 14-40 |  |
| **Gabapentin** | 9 (27) | 13-46 | 27 (38) | 26-50 |  | 11 (36) | 19-55 | 13 (26) | 14-40 |  |
| **Pregabalin** | 3 (9) | 2-24 | 10 (14) | 7-24 |  | 2 (7) | 1-21 | 9 (18) | 8-31 |  |
| **Lamotrigine** | 3 (9) | 2-24 | 4 (6) | 2-14 |  | 5 (16) | 6-34 | 12 (24) | 13-38 |  |
| **Other** | 4 (12) | 3-28 | 12 (17) | 9-27 |  | 3 (10) | 2-26 | 10 (20) | 10-33 |  |
| **First-line** | 24 (73) | 55-87 | 36 (50) | 38-62 | .05^2^ | 24 (77) | 59-90 | 29 (57) | 42-71 | .10^2^ |
| **Second-line** | 14 (42) | 26-61 | 38 (53) | 41-65 | .44^2^ | 17 (55) | 36-73 | 29 (57) | 42-71 | > .99^2^ |
| **One drug** | 24 (73) | 55-87 | 56 (78) | 66-87 |  | 19 (61) | 42-78 | 32 (63) | 48-76 |  |
| **Two drugs** | 8 (24) | 11-43 | 15 (21) | 12-32 |  | 10 (33) | 17-51 | 16 (31) | 19-46 |  |
| **Three or more drugs** | 1 (3) | 0-16 | 1 (1) | 0-8 |  | 2 (7) | 1-21 | 3 (6) | 1-16 |  |
| These tables summarize medication use in the MVD (S2A) and medical (S2B) cohorts, stratified by sex at baseline and after 5 years. Data include proportions and 95% confidence intervals for medication status, specific drugs, number of drugs used, and classification as first-line (carbamazepine or oxcarbazepine) or second-line (gabapentin, pregabalin, or lamotrigine). Statistical comparisons between men and women were performed using Fisher’s Exact Test^1^ or Pearson’s Chi-square test^2^. Abbreviations: MVD; microvascular decompression, CI; confidence interval. | | | | | | | | | | |

| **Supplementary Table 3a. Quality of Life** | | | |
| --- | --- | --- | --- |
|  | **Medical cohort** | **MVD cohort** | ***p-*value** |
| **Symptoms of depression,** N (%; 95% CI) | 49 (39; 31-48) | 29 (30.9; 22-41) | .26^1^ |
| **Overall satisfaction^a^,** median (IQR) | - | 7.0 (1.75) |  |
| **Treatment efficacy satisfaction,** median (IQR) | 7.0 (2.0) | - |  |
| **Side effects satisfaction,** median (IQR) | 6.0 (3.0) | - |  |
| **TN knowledge satisfaction,** median (IQR) | 6.0 (2.0) | - |  |
| Comparison of self-reported symptoms of depression and satisfaction with treatment aspects between medical and MVD groups. Satisfaction rated on a 0–7 Likert scale. Group comparisons performed using Pearson’s Chi-square test. ^a^Now vs. before MVD.  Abbreviations: MVD, microvascular decompression; CI, confidence interval; IQR, interquartile range; TN, trigeminal neuralgia. | | | |

| **Supplementary Table 3b. Multidisciplinary Team** | | | |
| --- | --- | --- | --- |
|  | **Medical cohort** | **MVD cohort** | ***p-*value** |
| **Nurse,** N (%; 95% CI) | 55 (42; 33-51) | 28 (30; 21-40) | .08^1^ |
| **Physiotherapist,** N (%; 95% CI) | 6 (5; 2-10) | 1 (1; 0-6) | .24^2^ |
| **Psychologist,** N (%; 95% CI) | 10 (8; 4-14) | 5 (5; 2-12) | .59^2^ |
| Proportion of patients reporting contact with a nurse, physiotherapist, or psychologist during treatment, stratified by cohort. Includes 95% confidence intervals and p-values from Pearson’s Chi-square^1^ or Fisher’s exact^2^ tests.  Abbreviations: MVD, microvascular decompression; CI, confidence interval. | | | |
